# Behavior of addicted patients during the COVID-19 pandemic

**DOI:** 10.1192/j.eurpsy.2022.2132

**Published:** 2022-09-01

**Authors:** Z. Bencharfa, Y. Amara, L. Tbatou, F. El Omari

**Affiliations:** University Hospital Center Ibn Sina, Ar-razi Psychiatric Hospital, Salé, Morocco

**Keywords:** Covid-19, Addiction, pandemic

## Abstract

**Introduction:**

The COVID-19 pandemic led governments to take a number of restrictive measures, which had an impact on the consumption of psychoactive substances among the world population.

**Objectives:**

The present study, carried out by the Addictology Center of Ar-razi Hospital in Salé, aimed to evaluate the behavior of addicted patients followed in ambulatory care, during the Covid-19 pandemic.

**Methods:**

We conducted a cross-sectional study with 128 patients, through a questionnaire assessing sociodemographic factors, psychiatric history, type and quantity of substances used during the pandemic, and withdrawal attempts.

**Results:**

The primary substance used was tobacco, followed by Cannabis, alcohol, hypnotics, and then Cocaine.

63% of patients reported an increase in their consumption during the pandemic, 64% started new substances, mainly Cannabis, followed by organic solvents.

The monthly amount spent by our patients varied from 300 to 40,000 dhs/month, the source of this amount was legal in 92.2% of the cases, 43.8% had already been incarcerated or taken into custody as a result of this consumption.

78% of our patients had already tried to wean themselves off the drug, but only 39% were able to succeed.

**Conclusions:**

The pandemic had a profound effect on the incidence of substance use.

Confining the population has indeed reduced the transmission of the virus, but it is far from harmless for the mind.

**Disclosure:**

No significant relationships.

